# Investigation of a spatial coupling relationship between carbon emission performance and regional urbanization in China

**DOI:** 10.1371/journal.pone.0222534

**Published:** 2019-09-30

**Authors:** Huimin Zhou, Pei Wang, Chengxin Wang, Xiangzheng Deng, Kai Liu

**Affiliations:** 1 Collaborative Innovation Center of Human-Nature and Green Development in Universities of Shandong, College of Geography and Environment, Shandong Normal University, Jinan, Shandong, China; 2 Institute of Geographic Science and Natural Resources Research, Chinese Academy of Sciences, Beijing, China; 3 University of Chinese Academy of Sciences, Beijing, China; 4 Center for Chinese Agricultural Policy, Chinese Academy of Sciences, Beijing, China; Shandong University of Science and Technology, CHINA

## Abstract

In light of the problem of environmental pollution caused by fossil fuel combustion, and its association with rapid urbanization, China is grappling with the question of how to reduce carbon emissions through more efficient energy consumption while simultaneously advancing its economic development. We applied a directional distance function to estimate the carbon emission performance of 30 provinces in China during the period 2000–2016. We selected an index system to assess urbanization processes in these provinces and conducted a spatial analysis to investigate the relationship between urbanization and carbon emission performance. We obtained the following results. First, the carbon emission performance of the eastern region, valued at 0.853, was relatively higher than the corresponding values of 0.810, 0.804, and 0.843 in the central, western, and northeastern regions, respectively. However, during this period, disparities among provinces increased. Second, the average urbanization value for each province showed an upward trend during the study period, and urbanization assumed a “striped” spatial agglomeration pattern. A third finding was that carbon emission performance and urbanization demonstrated a relationship of positive spatial dependence. The average value of their coordinated coupling indicated an upward trend, with an annual increase of 0.85%. Last, we found that efforts to reduce carbon emissions that are solely based on carbon emission performance do not yield reliable results. Accordingly, measurements of urbanization values can enable more detailed differentiation. In conclusion, reasonable measures should be implemented to improve carbon emission performance and urbanization that are in alignment with the actual situation within a given region.

## Introduction

The phenomenon of global warming, which has been associated with an increase in the average surface temperature by 1°C since 1880, is one of the greatest challenges currently facing governments and scientists worldwide [1]. There are many factors that contribute to global warming, which cannot be attributed solely to natural processes; anthropogenic activities, especially carbon dioxide (CO_2_) emissions, are also causative factors [2]. China is currently undergoing a process of rapid development and economic growth, entailing extensive urbanization and industrialization and, concomitantly, the consumption of vast amounts of energy resources, leading to increased CO_2_ emissions. Available statistics reveal that China’s consumption of energy amounted to 4.05 billion tons in 2016, with an increase of 25.8% compared with consumption levels in 2000. In 2009, China surpassed the United States as the world’s largest energy consumer and CO_2_ emitter, leading to the implementation of actions and measures to address climate change and promote sustainable development through a reduction in CO_2_ emissions [3].

The Chinese government is committed to implementing a CO_2_ reduction program, with a target of attaining peak CO_2_ emissions before 2030 and reducing total CO_2_ emissions by approximately 60–65% in 2030 compared with the total emission amount in 2005 [4]. The conventional pathway of accelerating the economy at the expense of natural habitats is being superseded by a roadmap for achieving a sustainable low-carbon economy. This roadmap has been proposed and prioritized in China’s National New Urbanization Plan (2014–2020) for achieving the country’s CO_2_ emissions goal [5]. The implementation of diverse acts and measures that are conducive to the pursuit of a low-carbon economy during the current period of rapid urbanization is recommended. The transition to this pathway mainly hinges on the adoption of new technologies and an optimal path for achieving carbon reduction. Thus, the implementation of technologies, such as carbon capture and storage, to prevent the release of CO_2_ into the atmosphere is required. These approaches are effective but much more expensive than the current carbon abatement technologies [6]. A second approach, which is more suitable and efficient for achieving carbon reduction, is to prohibit CO_2_ emissions from the onset. Nearly 87% of CO_2_ emissions are caused by the burning of fossil fuels worldwide. Consequently, the use of renewable energy derived from natural resources instead of fossil fuels is a more sustainable approach [3]. To achieve its target for 2030, the Chinese government is encouraging power plants to make their utmost efforts to generate electricity and heat using renewable resources such as sunlight, wind, and water. A third universally preferred method of achieving the above target is to improve energy efficiency, which is also one of the most economical and convenient approaches for optimizing energy inputs. Thus, a study in which the energy efficiency of each Chinese province is calculated and energy-inefficient regions are identified would be highly pertinent.

Previous researchers who have attempted to calculate energy efficiency or carbon emission performances have applied either parametric or nonparametric approaches [7]. Whereas data envelopment analysis (DEA) is commonly applied as a non-parametric tool, it does not reflect the relationship between energy consumption and its outputs. Stochastic frontier analysis is an alternative parametric approach used to calculate carbon emission performance. Moreover, directional distance function, conceived as a concrete framework for gauging producers’ performances in settings entailing multilateral production with multiple outputs, has been proposed as another calculative method [8]. From a comparative perspective, each of the above approaches, namely stochastic frontier analysis and directional distance function, have their own advantages and are convenient to implement and effective for calculating efficiency. Directional distance function, which entails the use of a complex panel dataset and multilevel model, is considered more effective than DEA [9].

Carbon emission performance can be understood as a synthesis of all relevant indicators such as energy consumption, economic activity and carbon dioxide emission, integrated within an overall index for the convenience of performance comparison [10]. Investigations of carbon emission performances have been more focused on the achievement of a balanced state between inputs and outputs in a developing region [11].The following scenarios can be considered: (1) high carbon emission performance with a high regional GDP output, low CO_2_ output, and low energy input; (2) high carbon emission performance with a relatively low regional GDP output, low CO_2_ output, and low energy input, reflecting a relatively high level of balance; (3) low carbon emission performance with a low regional GDP output, high CO_2_ output, and high energy input; and (4) low carbon emission performance with a relatively low regional GDP output, low CO2 output, and low energy input, reflecting a relatively low level of balance. Accordingly, the inclusion of an indicator for conducting a specific assessment of the degree of development of regional carbon emission performance is required to enable the proposal of more targeted measures.

China is undergoing rapid urban development. Consequently, there is an urgent need to reduce CO2 emissions associated with this process. Extensive research has been conducted on the relationship between carbon emission performance and urbanization, with the aim of improving the former. However, the results of studies that have examined this relationship differ because of variations relating to the research field, stage, and area. Thus, three key findings are evident within the literature. The first is that the level of urbanization is positively related to carbon emissions performance. Thus, it is posited that an improvement in the urbanization level will lead to the advancement of science and technology [12], the transformation of the industrial structure, and the improvement of labor quality [13]. All of these shifts will significantly contribute to the improvement of the carbon emission performance of regions. The second is that urbanization has a negative effect on carbon emissions performance because of its scale and agglomeration effects. Some scholars contend that the development of urbanization will prompt a dramatic increase in urban population and in energy consumption, which will significantly constrain the improvement of carbon emission efficiency [14, 15]. Third, many researchers have posited that the relationship between urbanization and carbon emissions demonstrates an inverse U-shaped relationship or Environmental Kuznets Curve. That is to say, with the continued advancement of urbanization, carbon emissions will peak and then gradually decline [16, 17].

Despite the lack of consensus, the existing literature reveals the existence of a close relationship between urbanization and carbon emissions. A comprehensive index that includes population urbanization, spatial urbanization (land urbanization), and economic and social urbanization is required for the study of urbanization. Population urbanization implies a large influx of people entering a city, which leads to increasing pressures on housing and transportation, and on the environment, that consequently impact on carbon emission performance [18, 19]. Economic and social urbanization entail a switch from agricultural activities to non-agricultural activities. While this process generates economic benefits, it may result in an increase in secondary industries as well as energy consumption, which could also affect the carbon emission performance. The third type of urbanization, which is spatial, is mainly reflected in the transformation of urban land types and their quantities. Variations in energy consumption relating to different types of land will produce differential economic benefits that will inevitably affect carbon emission performance. Thus, the construction of a comprehensive urbanization index system is essential, enabling a more scientific and detailed assessment of carbon emission performance in relation to varying levels of urbanization. The carbon emission performance of a region can be assessed more accurately by spatially mapping the development stage of urbanization on to carbon emissions. Consequently, countermeasures that are more consistent with the actual development stage can be proposed on the basis of the actual situation within a region.

Our aim was to analyze the relationship between urbanization and carbon emission performance in China using data collected for each province during the period 2000–2016. We first applied the directional distance function to calculate carbon emission performance. Subsequently, we obtained the spatial pattern of the carbon emission performance of each province. Next, we calculated the urbanization value for each province using the index system established within existing studies. Last, we analyzed the spatial autocorrelation between the carbon emission performance and urbanization. Our focus in this study was on the spatial relationship between carbon emission performance and urbanization. Accordingly, we applied a model to explore the extent of balanced coordination between them and recommended a scientific development approach that would account for differences in regional development.

## Methodology

### Directional distance function

The directional distance function was used to estimate the efficiency of energy consumption and the carbon emission performances of 30 provinces in China during the period 2000–2016. An effective measure for gauging producers’ performances within a setting entailing multilateral production and multiple outputs has previously been developed [8]. This measurement of productivity encompasses desirable as well as undesirable production outputs. Applying this theoretical framework, we measured the efficiency of energy consumption and carbon emission performance in the course of the urbanization process along with both economic development (a desirable output or “good”) and CO_2_ emissions (an undesirable output or “bad”). Accordingly, the output vector in our study can be expressed as $(y,b)\in R_{+}^{N}$, where the desirable output is GDP (*y*) and the undesirable output is CO_2_ emissions (b) generated by the input vector, capital (*k*), labor (*l*), and energy use (*e*) [20]. Thus, the production function *S* can be expressed as follows:

S(k,l,e)={(x,y,b):xcanproduce(y,b)} (1)

where $x=(k,l,e)\in R_{+}^{N}$. A specific assumption is that the desirable output, *y*, and the undesirable output, *b*, are jointly produced. This assumption is termed a weak disposability property, indicating that the abatement of the undesirable output occurs at the expense of economic growth. Thus, CO_2_ reduction is proportionate to the reduction of the GDP, with the multiplier *θ* representing the proportion of the abatement [21]. Applying Shephard’s definition, we interpreted weak disposability as follows:

1. If outputs are weakly disposable, then (*x*,*y*,*b*)∈*S* while 0≤*θ*≤1 and (*x*,*θy*,*θb*)∈*S*. This implies that a given set of inputs can generate any kind of contraction of the assembly of “goods” and “bads”.
2. If (*x*,*y*,*b*)∈*S* and *b* = 0, then *y* = 0. Accordingly, if no undesirable outputs are produced, then there will correspondingly be no desirable outputs.

We constructed our function as follows. Drawing on the original concept developed by Färe et al [8], we incorporated the idea of maximizing the desirable output and minimizing the undesirable output proposed by Wang et al [20]. The frontier can be expressed as follows:

$$D(k,l,e,y,b;g)=sup\{\lambda :(y+\lambda g_{y},b-\lambda g_{b})\}$$ (2)

where *g*_*y*_ denotes the direction of the expansion of the GDP, *g*_*b*_ denotes the direction of the contraction of CO_2_ emissions, *g* = (*g*_*y*_,*g*_*b*_) denotes the direction vector, and *λ* represents the maximum scale of expansion and contraction.

**Fig 1:**
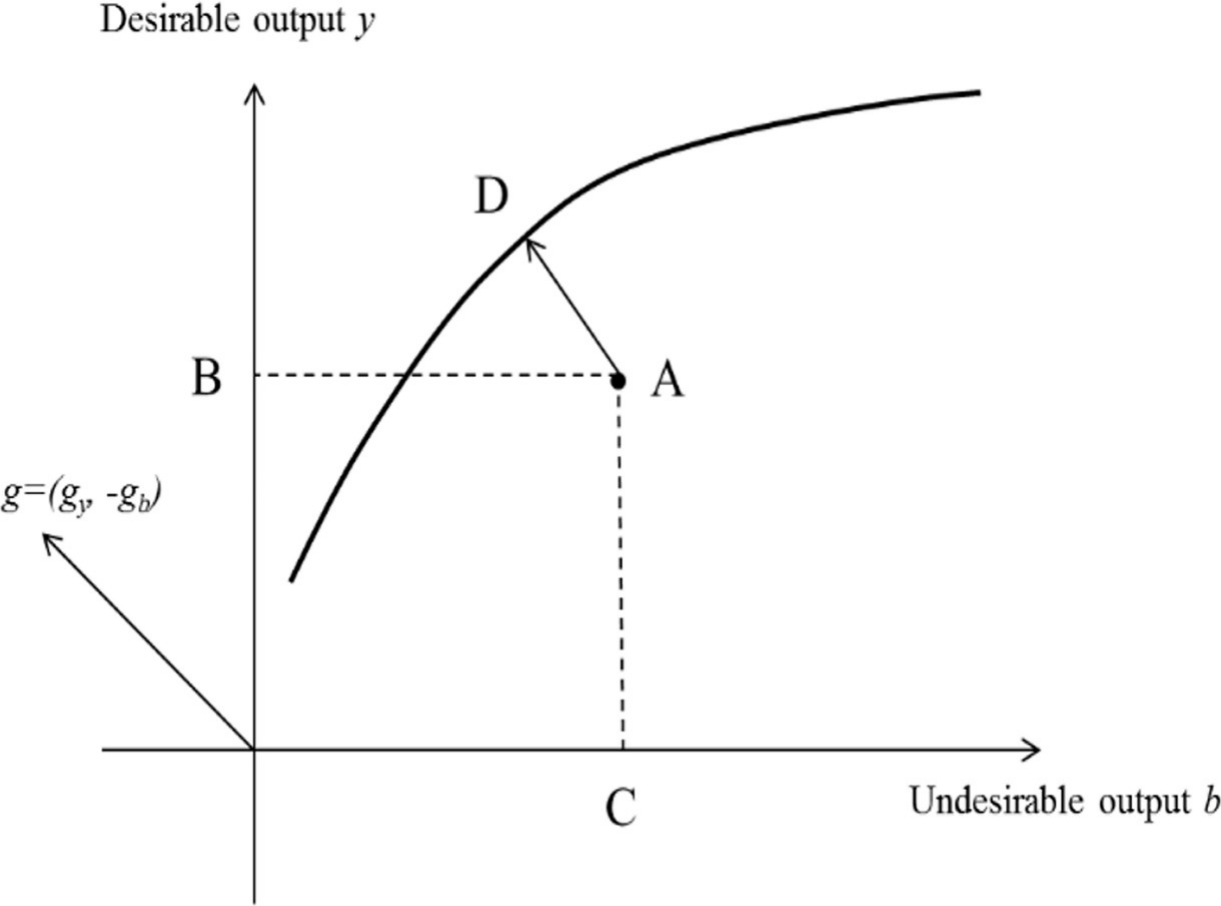
A depiction of the directional distance function.

Fig 1 depicts the directional distance function. It is assumed that *A*, representing a certain province of China, can be described by the production function, *S*(*k*,*l*,*e*), in which *OB* and *OC* represent the GDP and CO_2_ emissions in the province, respectively. To maximize the desirable output and minimize the undesirable output, point *A* should move toward *D*, which is located on the frontier. Thus, *D*(*k*,*l*,*e*,*y*,*b*;*g*) = *μ*, where *μ* refers to the technical inefficiency representing the distance between the frontier and *A* as the place of observation.

We included 18 primary indicators in our system for evaluating the quality of local environments to assess a region’s environmental performance. For the directional distance function, the minimal amount of CO_2_ emissions can be depicted as (1−*D*(*k*,*l*,*e*,*y*,*b*))×*b*, where Eq (3), that is, *E* = 1−*D*(*k*,*l*,*e*,*y*,*b*), expresses the carbon emission performance. Thus, a higher value for the carbon emission performance corresponds to a higher value of *E* estimated using the directional distance function.

### Estimation of directional distance function

We specifically included the Cobb-Douglas, translog, and quadratic production functions and adopted the translog form for our parametric estimation, which can be expressed as follows:

$$D(k,l,e,y,b)=\alpha_{0}+\alpha_{k}k+\alpha_{l}l+\alpha_{e}e+\alpha_{y}y+\alpha_{b}b+\alpha_{kl}kl+\alpha_{ke}ke+\alpha_{ky}ky+\alpha_{kb}kb+\alpha_{le}le+\alpha_{ly}ly+\alpha_{lb}lb+\alpha_{ey}ey+\alpha_{eb}eb+\alpha_{yb}yb+\frac{1}{2}\alpha_{kk}k^{2}+\frac{1}{2}\alpha_{ll}l^{2}+\frac{1}{2}\alpha_{ee}e^{2}+\frac{1}{2}\alpha_{yy}y^{2}+\frac{1}{2}\alpha_{bb}b^{2}+\upsilon$$ (4)

The weak disposability in this study entails the sacrifice of economic production to achieve CO_2_ reduction. If there is no economic output, then there is correspondingly no CO_2_ emission. The translation property of the directional distance function was expressed as follows:

$$D(k,l,e,y+\lambda g_{y},b-\lambda g_{b})=D(k,l,e,y,b)\;\mathrm{and}$$ (5)

D(k,l,e,y+b,0)=D(k,l,e,y,b)−b (6)

The following equation was derived from the translog form of the directional distance function (4):

$$D(k,l,e,y+b,0)=\alpha_{0}+\alpha_{k}k+\alpha_{l}l+\alpha_{e}e+\alpha_{y}(y+b)+\alpha_{kl}kl+\alpha_{ke}ke+\alpha_{ky}k(y+b)+\alpha_{le}le+\alpha_{ly}l(y+b)+\alpha_{ey}e(y+b)+\frac{1}{2}\alpha_{kk}k^{2}+\frac{1}{2}\alpha_{ll}l^{2}+\frac{1}{2}\alpha_{ee}e^{2}+\frac{1}{2}\alpha_{yy}{\left(y+b\right)}^{2}+\upsilon$$ (7)

Eq (8), which was used for the estimation, was generated by combining Eqs (4) and (6) with (7) as follows:

$$-b=\alpha_{0}+\alpha_{k}k+\alpha_{l}l+\alpha_{e}e+\alpha_{y}(y+b)+\alpha_{kl}kl+\alpha_{ke}ke+\alpha_{ky}k(y+b)+\alpha_{le}le+\alpha_{ly}l(y+b)+\alpha_{ey}e(y+b)+\frac{1}{2}\alpha_{kk}k^{2}+\frac{1}{2}\alpha_{ll}l^{2}+\frac{1}{2}\alpha_{ee}e^{2}+\frac{1}{2}\alpha_{yy}{\left(y+b\right)}^{2}+\upsilon -\mu$$ (8)

### Evaluation of regional urbanization

We evaluated the level of urbanization by applying the following four steps. After standardizing the raw data, we calculated the standard entropy, determined weights, and assessed the level of urbanization [22]. The original data were incorporated into a standard dataset within the range 0–1 as follows:

$$X_{ij}=\frac{x_{ij}-x_{jmin}}{x_{jmax}-x_{jmin}}$$ (9)

where, *i* is the year of observation, (*i* = 2000, 2001,…2016), *j* denotes the number of indicators, (*j* = 1, 2, 3…15), *xij* is the original data for each indicator for the period 2000–2016, *Cij* denotes standardized data for each indicator, *x*_*jmin*_ is the smallest value of index *j*, and *x*_*jmax*_ denotes the greatest value of index *j*.

Eqs (10) and (11) express the standard entropy (*e*_*j*_) process.

$$e_{j}=\frac{Y_{ij}\times {lnY}_{ij}}{lnm}$$ (10)

$$Y_{ij}=\frac{c_{ij}}{\sum_{i=1}^{m}c_{ij}}$$ (11)

where *m* is the total number of indicators and *e*_*j*_ denotes standard entropy.

As a third step, we calculated the weight of each indicator (*H*_*i*_) for the period 2000–2016 in relation to entropy as follows:

$$H_{i}=\frac{1-e_{j}}{\sum_{j=1}^{mn}\left(1-e_{j}\right)}$$ (12)

where *n* denotes the number of years.

Last, the urbanization of each region (*Z*_*i*_) was calculated as the sum of the weights of the different indicators multiplied by the standardized value of indicators, expressed below:

$$Z_{i}=\sum_{i=1}^{m}H_{i}b_{ij}$$ (13)

### A spatial analysis of the relationship between carbon emission performance and urbanization

We performed a spatial analysis to identify the spatial dependence and heterogeneity of the samples. Spatial autocorrelation, which is at the core of spatial dependence, is conducted to identify whether a correlation exists between an observed sample and its neighbor. It is also conducted to measure the degree of spatial agglomeration [23]. Global as well as local spatial autocorrelation analyses are commonly conducted. Global spatial autocorrelation is performed to analyze the distribution characteristics of the spatial data at the level of the overall study area. Global Moran’s *I* (*I*_*g*_) is a widely used indicator in this type of analysis. If a vector *x* = {*x*_1_,*x*_2_,…,*x*_*n*_}, then

$$I_{g}=\frac{n\sum_{i=1}^{n}\sum_{j=1}^{n}w_{ij}(x_{i}-\bar{x})(x_{j}-\bar{x})}{\sum_{i=1}^{n}\sum_{j=1}^{n}w_{ij}\sum_{i=1}^{n}{\left(x_{i}-\bar{x}\right)}^{2}}=\frac{\sum_{i=1}^{n}\sum_{j=1}^{n}w_{ij}(x_{i}-\bar{x})(x_{j}-\bar{x})}{S^{2}\sum_{i=1}^{n}\sum_{j=1}^{n}w_{ij}}\;\mathrm{and}$$ (14)

$$\bar{x}=\frac{1}{n}\sum_{i=1}^{n}x_{i},\;S^{2}=\frac{1}{n}\sum_{i=1}^{n}{\left(x_{i}-\bar{x}\right)}^{2}$$ (15)

where *n* is the number of regions in the overall study area, *w*_*ij*_ is the spatial weight (if regions i and j are neighbors, then *w*_*ij*_ = 1; if they are not neighbors, then *w*_*ij*_ = 0).

By contrast, local spatial autocorrelation analysis is conducted to determine a local region’s relationships with adjacent regions. A map of local indicators of spatial association (LISA) is commonly used to depict spatial agglomeration and to enable the measurement of the heterogeneity of a local region and its neighbors. A LISA map enables the assessment of High–High, Low–Low, High–Low and Low–High spatial agglomerations. The Local Moran’s *I* (*I*_*l*_) was expressed as follows:

$$I_{i}=\frac{n^{2}(x_{i}-\bar{x})\sum_{j=1}^{n}w_{ij}(x_{j}-\bar{x})}{\sum_{j=1}^{n}w_{ij}\sum_{j=1}^{n}{\left(x_{j}-\bar{x}\right)}^{2}}\;\mathrm{and}$$ (16)

$$\sum_{i=1}^{n}I_{i}=n\times I_{g}$$ (17)

### Model for coordinating coupling

The concept of coupling, which is derived from physics, refers to a phenomenon in which two (or more) systems interact with each other [24]. We defined carbon emission performance and urbanization quality as two systems, and investigated their correlation and influence on each other using the following equations:

$$C=\sqrt{\frac{R_{1}R_{2}}{{\left(\frac{R_{1+}R_{2}}{2}\right)}^{2}}}$$ (18)

$$D=\sqrt{C\times T}$$ (19)

$$T=\alpha R_{1}+\beta R_{2}$$ (20)

where *C* is the coupling factor (*C*∈[0,1]). A higher degree of coupling between these two systems, corresponds to a closer approximation of the value of *C* to 1. When the value of *C* is 0, carbon emission performance is not connected with urbanization. *R*_1_ denotes the system of carbon emission performance, and *R*_2_ denotes the system of urbanization. *D* denotes the result of the coordination model, *C* denotes the comprehensive index of two systems, and *α* and *β* denote the respective contributions of carbon emission performance and urbanization, with *α*+*β* = 1. Table 1 presents the results for the degree of coupling between carbon emission performance and urbanization with reference to previously reported findings [25, 26].

**Table 1:** Categories relating to the degree of coupling between carbon emission performance and urbanization.

Primary development stages	Secondary development stages		Tertiary development stages	
Unbalanced development period	0<*D*< = 0.3	Seriously unbalanced development		
	0.3< = *D*< = 0.5	Unbalanced development		
Transition period	0.5< = *D*< = 0.7	Barely balanced development	0.5< = *D*< = 0.6	Slightly balanced development
			0.6< = *D*< = 0.7	Low-level balanced development
Balanced development period	0.7< = *D*< = 1	Balanced development	0.7< = *D*< = 1	Basic balanced development
			0.7< = *D*< = 1	High-level balanced development
			0.7< = *D*< = 1	Superior balanced development

## Data sources

### Dataset for estimating carbon emission performance

As noted earlier, we collected data on energy consumption, labor, capital stock, and the GDP of each province from the statistic yearbook of China for the period 2001–2017 [27]. Table 2 provides statistical descriptions of input and output variables for the directional distance function.

**Table 2:** Statistical descriptions of input and output variables for the directional distance function.

Variable	Unit	Mean	Std. Dev	Min	Max
Capital stock (k)	0.1 RMB Billion	6627	6692	126	36983
Labor (L)	10 thousands people	2503	1676	275.5	6905
Energy (E)	10 thousand tons of standard coal	10766	7702	480	38899
GDP (y)	0.1 RMB Billion	7897	7400	264	45995
CO_2_ emission (b)	10 thousand tons	47881	38577	1175	220807

We applied constant prices for 1952 to exclude the effects of inflation on capital stock and the GDP. We included 30 provinces in our dataset. Tibet was excluded because of the lack of available data. Moreover, we converted the consumption of 11 energy sources (coal, coke, oil, crude oil, gasoline, kerosene, diesel oil, fuel oil, liquefied petroleum gas, natural gas, and electricity) into consumption values for standard coal [28]. Table 3 shows the results for CO_2_ emission factors that were calculated using Eq (21), with data on different kinds of energy consumption and emission factors sourced from different sites [29–31].

We used the following formula for our calculation

$$b_{i}={\sum_{j}^{11}(E}_{ij}\times {LCV}_{ij}\times {CPC}_{ij}\times {COV}_{ij})\times \frac{44}{12}$$ (21)

where *b*_*i*_, *LCV*_*i*_, *CPC*_*i*_, and *COV*_*i*_ denote CO_2_ emissions, fuel calorific values, emission factors, and energy oxidation rates, respectively. The parameters applied in the formula were obtained from the energy statistics yearbook [32].

**Table 3:** CO_2_ emission factors.

Indicators	CO_2_ emission factor	Indicators	CO_2_ emission factor
coal(t/t)	2.640	kerosene(t/t)	3.152
coke(t/t)	2.852	diesel oil(t/t)	3.145
oil(t/t)	2.080	fuel oil(t/t)	3.047
crude oil(t/t)	1.921	LPG(t/t)	2.954
gasoline(t/t)	3.043	natural gas(t/10^4^ m^3^)	21.622

As Table 4 shows, the CO_2_ emission factor for electricity varied across different regions. All of the parameters shown in Table 4 were obtained from the energy statistics yearbook [32].

**Table 4:** Emission factors for electricity in different Chinese provinces.

Region	CO_2_ emission factor(kgCO_2_/kWh)	Region	CO_2_ emission factor(kgCO_2_/kWh)
China	0.6590	Henan	0.8063
Beijing	0.7757	Hubei	0.3526
Tianjin	0.8917	Hunan	0.5166
Hebei	0.8981	Chongqing	0.5744
Shanxi	0.8488	Sichuan	0.2475
Inner Mongolia	0.9292	Guangdong	0.5912
Shandong	0.8878	Guangxi	0.4948
Liaoning	0.7753	Guizhou	0.4949
Jilin	0.7214	Yunnan	0.3063
Heilongjiang	0.7970	Hainan	0.6855
Shanghai	0.6241	Shaanxi	0.7690
Jiangsu	0.7498	Gansu	0.5729
Zhejiang	0.6647	Qinghai	0.2323
Anhui	0.8092	Ningxia	0.7789
Fujian	0.5514	Xinjiang	0.7890
Jiangxi	0.6336		

### Index system for regional urbanization

Urbanization, by definition, describes a process whereby a rural population is transformed into an urban population, agricultural land is transformed into urban land, and agricultural activities are transformed into non-agricultural activities. Such radical shifts are bound to induce life-transforming social changes during this process. Therefore, in our study, urbanization not only means the urbanization of the population; it entails a comprehensive process of urbanization.

Urbanization reflects a process of economic and social development, population increase, and expansion of urban land use. To produce an accurate estimate of the urbanization of cites, we established a comprehensive index system, with reference to previously developed indexes [27, 31, 32]. These indexes were selected on the basis of a qualitative comparison of their importance for urbanization. In our study, urbanization covers demographic, economic, social, and spatial urbanization (see Table 5). Economic and social urbanization are considered basic forms of urbanization. They include per capita GDP, the proportion of the added value of secondary and tertiary industries to GDP, the total fixed asset investment per capita, the consumption level of residents per capita, the number of phones per 10,000 people, and the number of buses per 10,000 people. Demographic urbanization in this study refers to the performance of comprehensive urbanization, as indicated by the percentages of the nonagricultural population and of employment within secondary and tertiary industries. Spatial urbanization is indicated by the expansion of urban land and encompasses urban population density and the proportion of the total land area comprising built-up areas.

**Table 5:** The index system for urbanization.

Urbanization	Index^a^	References
Demographic urbanization	Percentage of nonagricultural population (%)	[28]
	Percentage of secondary and tertiary industry employment (%)	[28]
Spatial urbanization	Urban population density (persons/km^2^)	[33]
	Percentage of the total land area that is built-up area (%)	[28]
Economic urbanization	Per capita GDP (Yuan)	[33]
	Proportion of the added value of secondary and tertiary industry to GDP (%)	[28]
	Total fixed asset investment per capita (Yuan)	[34]
Social urbanization	Consumption level of the residents per capita (Yuan)	[28]
	Number of phones per 10,000 people	[28]
	Number of buses per 10,000 people	[34]

^a^ The data of the urbanization indexes are all from the China City Statistical Yearbook

(http://www.stats.gov.cn/tjsj/tjcbw/201806/t20180612_1604098.html)

## Results

### The carbon emission performance of each province

Table 6 presents estimates of the directional distance function. The negative sign of the term *ln(y) + ln(b)* indicates that higher CO_2_ emissions correspond to greater efficiency losses. By contrast, the efficiency loss is lower with an increase in the GDP. The terms *ln(K)* and *ln(L)* were statistically significant and their signs were negative, indicating that increased capital stock and labor inputs are associated with a lower loss in efficiency. However, the sign of the term *ln(E)* was positive, which means that the efficiency loss will be greater if the energy input is higher. The poor carbon emission performance of each province can be attributed to a deficiency in capital and labor inputs or to a surplus of energy inputs.

**Table 6:** Estimated results of model (8).

Variable	Coefficient	Z-statistic	Variable	Coefficient	Z-statistic
*Dependent variable*: *-ln(b)*					
*ln(K)*	-0.32	-1.72*	*ln(K)ln(L)*	0.049	1.61*
*ln(L)*	-0.85	-3.77***	*ln(K)ln(E)*	0.62	8.15***
*ln(E)*	0.84	1.57	*ln(K)(ln(y)+ ln(b))*	-0.41	-10.18***
*ln(y)+ ln(b)*	-0.27	-1.21	*ln(L)ln(E)*	0.67	10.43***
*(ln(K))*^*2*^	0.11	7.57***	*ln(L)(ln(y)+ ln(b))*	-0.29	-10.03***
*(ln(L))*^*2*^	-0.011	-0.47	*ln(E)(ln(y)+ ln(b))*	-0.87	-7.44***
*(ln(E))*^*2*^	0.22	1.37	*constant*	-2.47	-3.11***
*(ln(y)+ ln(b))*^*2*^	0.37	13.51***			
Log likelihood	256.33***		Likelihood-ratio test of sigma_u = 0	22.49***	

Asterisk symbols here refer to the significance of coefficients (it is represented by p), then

*- p<0.1

***-p<0.01.

**Fig 2:**
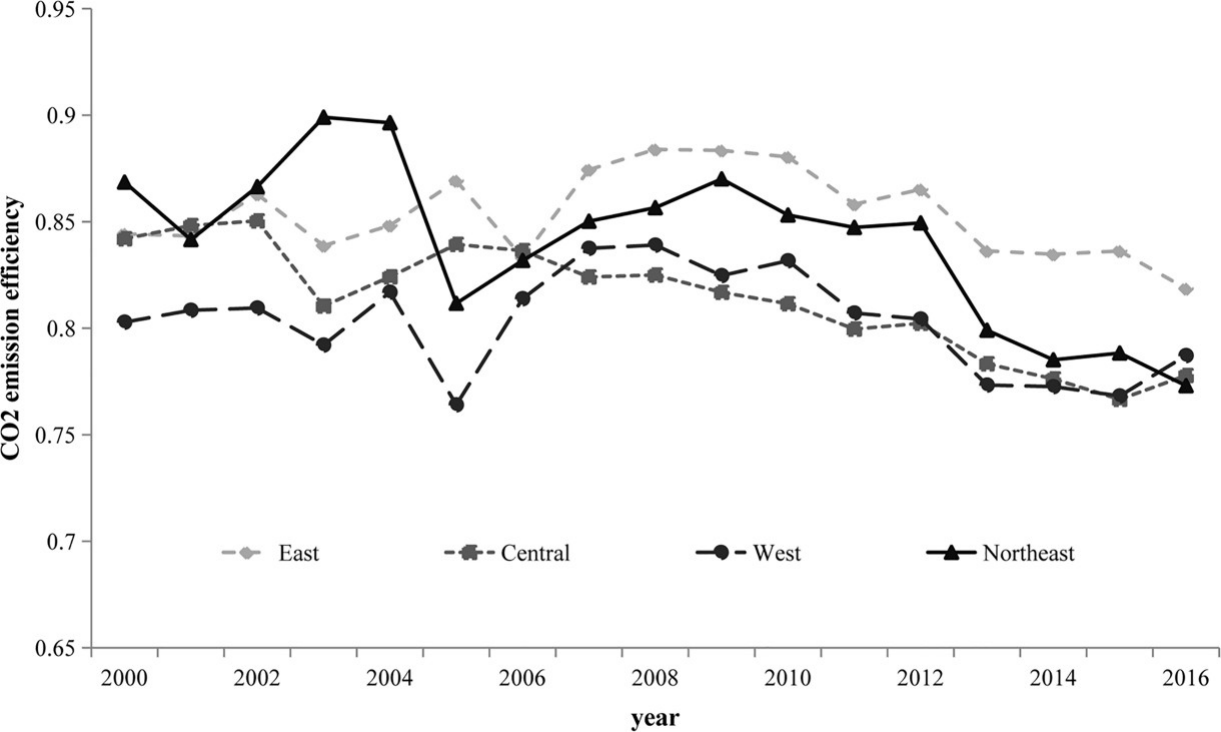
Carbon emission performances of the eastern, central, western, and northeastern regions of China during the period 2000–2016.

**Fig 3:**
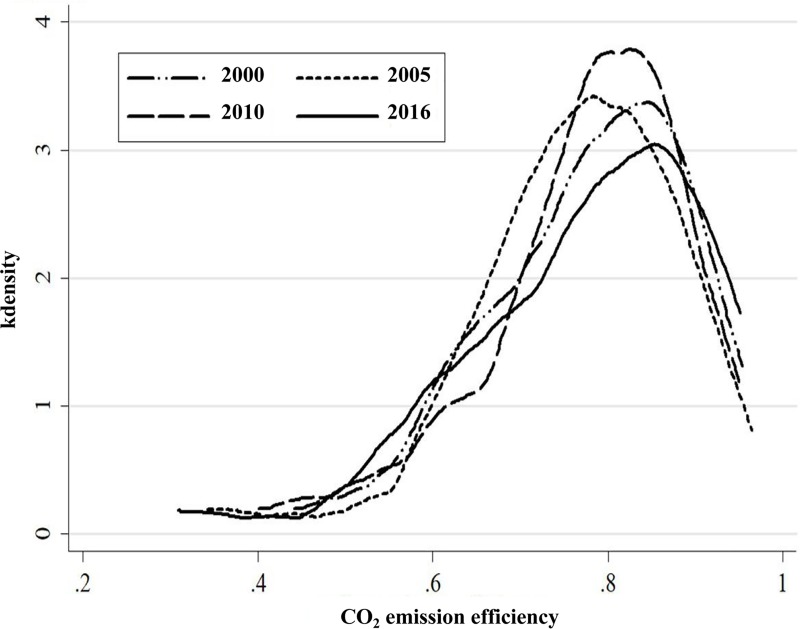
Kernel density of carbon emission performances in 2000, 2005, 2010, and 2016.

Table 7 shows the carbon emission performance of each province. The mean value of the carbon emissions performance of the eastern region was relatively higher than the mean values of the central, western, and northeastern regions, which were 0.81, 0.804, and 0.843, respectively. The curves depicted in Fig 2 directly demonstrate that the carbon emission performances of the eastern, central, and western regions declined slightly during the period 2000–2016 while dropping significantly in the northeastern region. Viewed in combination with the kernel density of the carbon emission performance shown in Fig 3, the curves for 2005 and 2016 were evidently flatter than those for other years. The efficiency values ranged from 0.48 to 0.98 in 2005 and from 0.58 to 0.98 in 2016. These figures suggest that the spatial disparities of energy efficiency were wider in 2005 and 2016 than they were in 2000 and 2010. Furthermore, the efficiency was lower than 0.72 in nine provinces in 2016, compared with the values in four provinces that were lower than 0.72 for each of the years 2000, 2005, and 2010. Thus, a large proportion of energy consumers demonstrated relatively lower energy efficiency in 2016. Of these consumers, some should be eliminated, and more energy consumers should make concentrated efforts to improve their energy efficiency in line with current input levels. In a context of rapid economic development and urbanization, it has become imperative to upgrade industries and optimize their structures to improve the quality of production and carbon emission performances in China.

**Table 7:** Carbon emission performances of 30 Chinese provinces during the period 2000–2016.

	2000	2005	2010	2016	Mean
**East region**	**0.845**	**0.867**	**0.878**	**0.822**	**0.853**
Beijing	0.72	0.84	0.96	0.98	0.87
Tianjin	0.82	0.80	0.91	0.92	0.86
Hebei	0.76	0.92	0.92	0.85	0.87
Shanghai	0.83	0.91	0.95	0.91	0.90
Jiangsu	0.81	0.79	0.83	0.75	0.79
Zhejiang	0.92	0.88	0.82	0.86	0.86
Fujian	0.98	0.96	0.95	0.96	0.94
Shandong	0.75	0.80	0.79	0.65	0.77
Guangdong	0.91	0.91	0.95	0.69	0.91
Hainan	0.95	0.88	0.70	0.65	0.76
**Central region**	**0.813**	**0.818**	**0.815**	**0.783**	**0.81**
Shanxi	0.56	0.72	0.77	0.71	0.71
Anhui	0.75	0.76	0.70	0.67	0.72
Jiangxi	0.90	0.80	0.78	0.77	0.80
Henan	0.78	0.74	0.72	0.69	0.74
Hubei	0.92	0.97	0.97	0.97	0.96
Hunan	0.97	0.92	0.95	0.89	0.93
**West region**	**0.803**	**0.763**	**0.818**	**0.787**	**0.804**
Inner Mongolia	0.64	0.82	0.76	0.60	0.70
Guangxi	0.93	0.87	0.78	0.75	0.83
Chongqing	0.79	0.90	0.94	0.95	0.92
Sichuan	0.88	0.91	0.95	0.97	0.93
Guizhou	0.62	0.61	0.73	0.68	0.66
Yunnan	0.88	0.83	0.83	0.87	0.85
Shaanxi	0.78	0.57	0.64	0.58	0.66
Gansu	0.64	0.64	0.81	0.83	0.76
Qinghai	0.95	0.49	0.97	0.86	0.90
Ningxia	0.84	0.77	0.68	0.66	0.70
Xinjiang	0.88	0.98	0.91	0.91	0.93
**Northeast region**	**0.867**	**0.813**	**0.853**	**0.773**	**0.843**
Liaoning	0.94	0.87	0.95	0.81	0.91
Jilin	0.82	0.78	0.80	0.75	0.81
Heilongjiang	0.84	0.79	0.81	0.76	0.81

**Fig 4:**
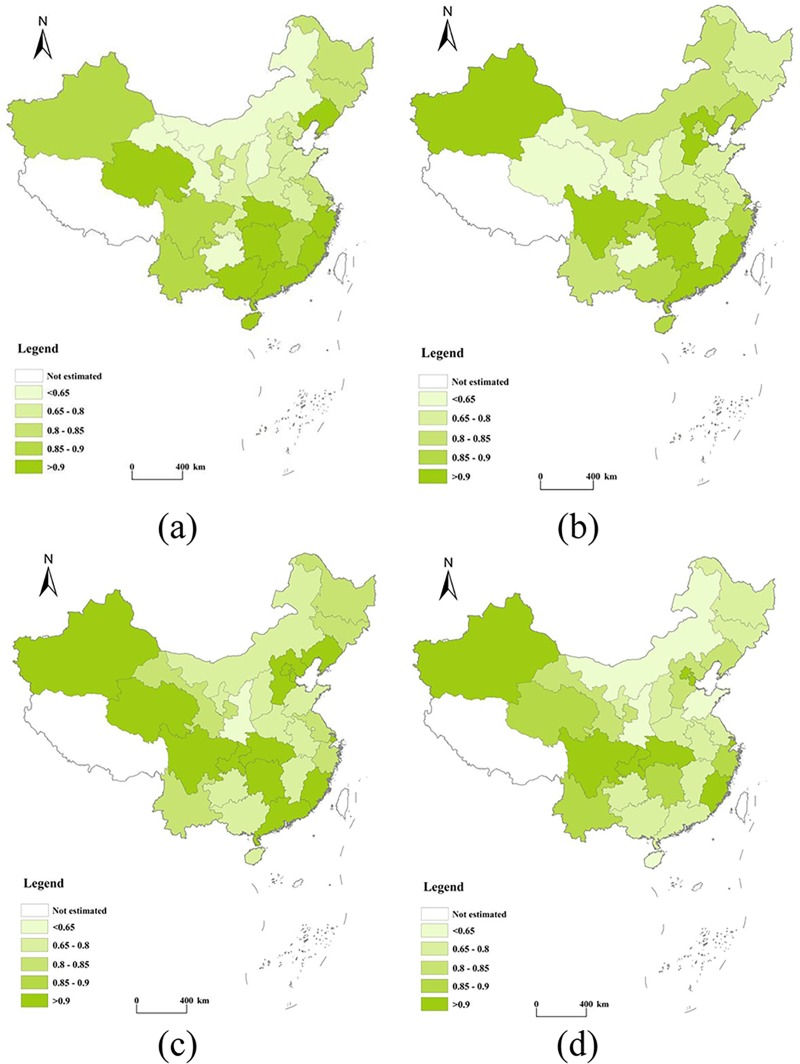
A spatial analysis of carbon emission performances of provinces in China in 2000 (a), 2005 (b), 2010 (c), and 2016 (d).

Fig 4 shows the spatial distribution of carbon emission performances in 2000, 2005, 2010, and 2016. On average, the efficiencies of the following provinces were higher than those of others during the period 2000–2016: Beijing (0.87), Hebei (0.87), Shanghai (0.90), Fujian (0.94), Guangdong (0.91), Hubei (0.96), Hunan (0.93), Chongqing (0.92), Sichuan (0.93), Qinghai (0.90), Xinjiang (0.93), and Liaoning (0.91). In 2016, efficiency was relatively higher in Beijing (0.98), Tianjin (0.92), Shanghai (0.91), Fujian (0.96), Hubei (0.97), Hunan (0.89), Chongqing (0.95), Sichuan (0.97), and Xinjiang (0.91). These findings indicate an expansion in inefficient energy consumption in Hebei, Guangdong, Qinghai, and Xinjiang, as a countertrend to that of an improvement in the average carbon emission performance. By comparison, the performances of Shanxi (0.71), Anhui (0.72), Inner Mongolia (0.70), Guizhou (0.66), Shaanxi (0.66), and Ningxia (0.70) were lower during the same period.

### Regional urbanization

**Fig 5:**
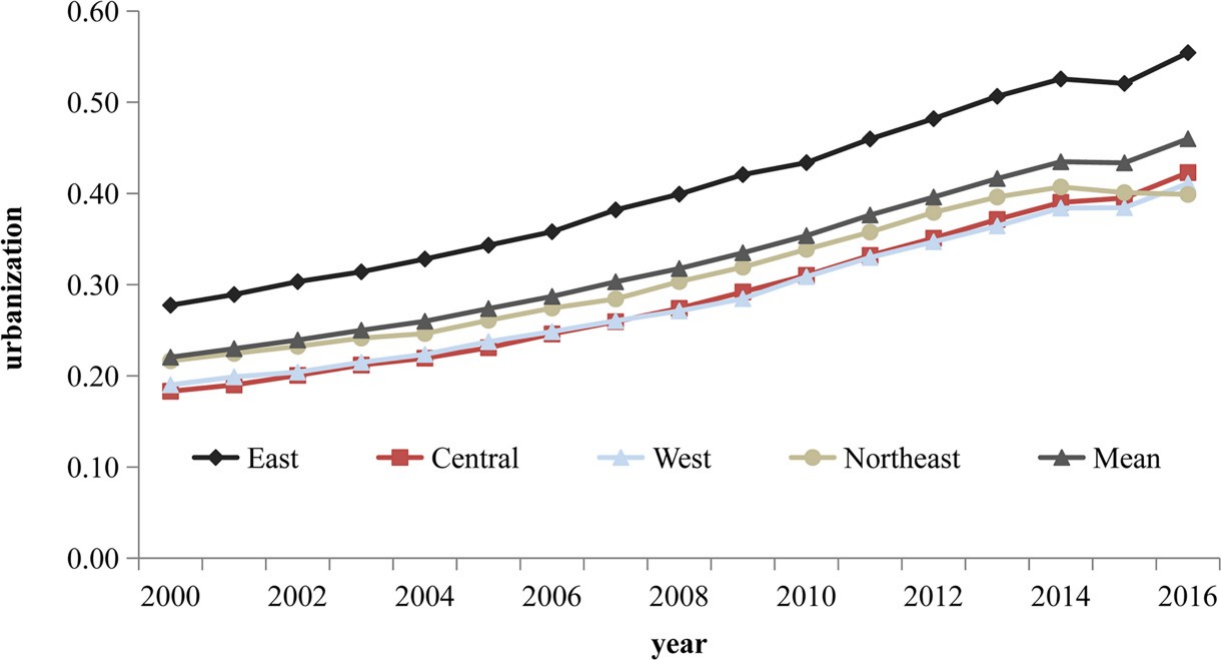
Mean values of urbanization for the eastern, central, western, and northeastern regions of China during the period 2000–2016.

As Fig 5 shows, the average urbanization value for each province showed an upward trend during the period 2000–2016, rising from 0.22 in 2000 to 0.46 in 2016, with an average annual growth rate of 6.429%. This finding indicates that urbanization demonstrated an overall growth trend, with a rapid increase in momentum. The mean value of urbanization in the eastern region was 0.406, exceeding the values obtained for the central, western, and northeastern regions, which were 0.287, 0.286, and 0.311, respectively. The eastern region of China has always been at the forefront of economic development. This is reflected in the region’s economic agglomeration, labor quality, and urban expansion, which far outpace those of other regions. Benefiting from the influence of national policies, urbanization in the northeastern region approximates the national average. The central and western provinces lag behind other regions in terms of urbanization as a result of topographical, historical-cultural, and societal influences. However, there has been an accelerating trend in the urban development of the central and western regions since 2015.

**Fig 6:**
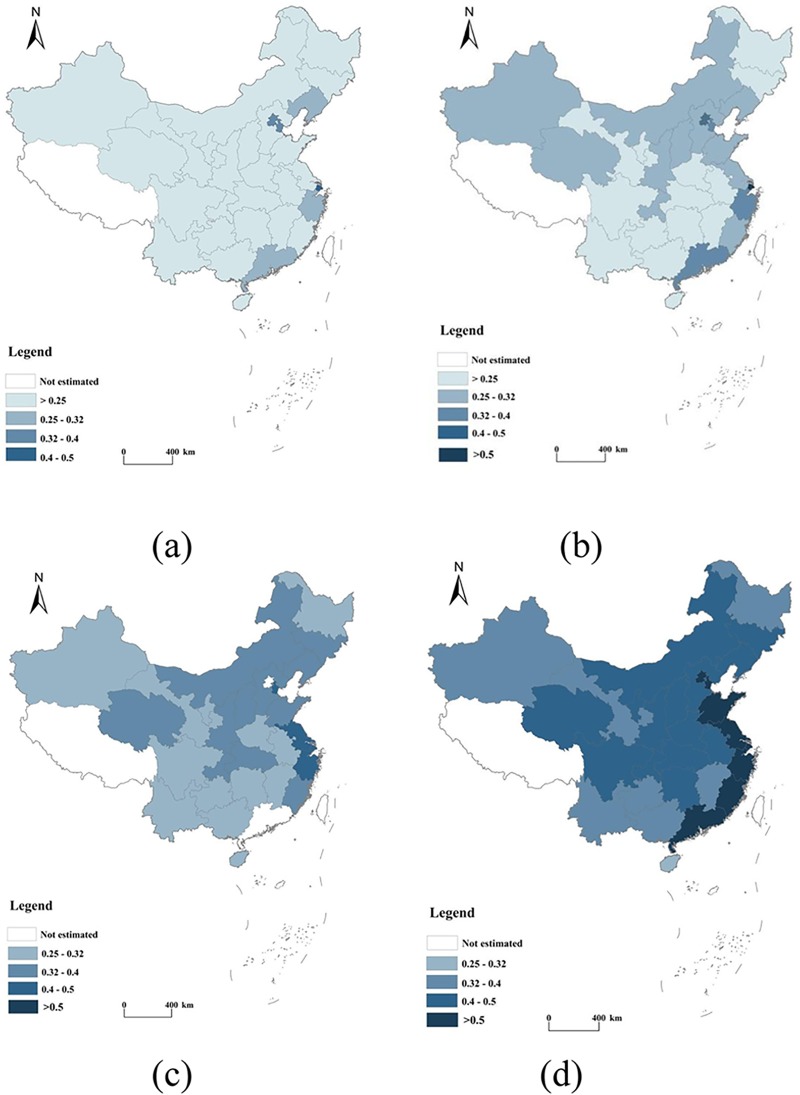
The spatial distribution of urbanization in 2000 (a), 2005 (b), 2010 (c), and 2016 (d).

Fig 6 shows that the spatial pattern of urbanization in the 30 provinces examined in the study evidenced a “striped” pattern of agglomeration. We further categorized this pattern in light of the results of our calculations. In this figure, a darker shade corresponds to a higher degree of urbanization. The higher values were concentrated along the eastern coast in Shanghai (0.562), Beijing (0.522), Tianjin (0.461), Guangdong (0.447), and Jiangsu (0.416) and were much higher, on average, than those of other provinces. The values for Liaoning, Shandong, Fujian, Qinghai, Hebei, Inner Mongolia, and Shanxi ranged between 0.278 and 0.362 and were categorized within a second tier, reflecting a relatively high degree of urbanization. Thus, the quality of urbanization in provinces with urbanization values below 0.25, namely, Henan (0.227), Gansu (0.238), Guangxi (0.29), Guizhou (0.242), and Yunnan (0.249) was relatively low compared with that of other provinces.

### The spatial relationship between carbon emission performance and regional urbanization

#### Spatial autocorrelation analysis

**Fig 7:**
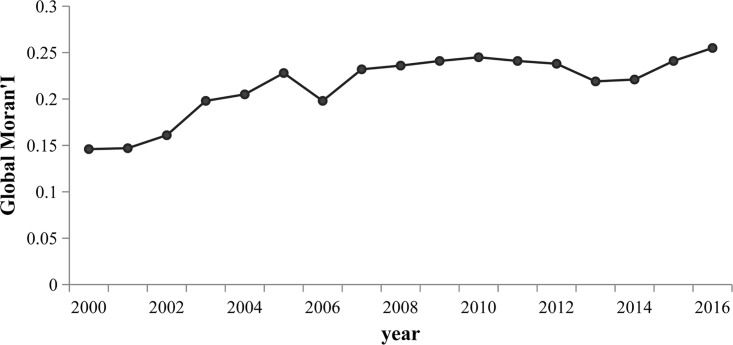
The change trend for values of the *Global Moran’s I* indicator for carbon emission performances and urbanization in China during the period 2000–2016.

We conducted a dependence analysis using spatial data on carbon emission performances and urbanization for 30 provinces in China to obtain the value of Global Moran’s I indicator during the period 2000–2016. Fig 7 shows changes in the value of this indicator for carbon emission performance and urbanization in China. The Global Moran’s I indicator values for carbon emission performance and urbanization ranged between 0.145 and 0.255 from 2000 to 2016. These values indicate that there was weak spatial dependence between these two indicators, with spatial agglomeration gradually becoming stronger during this period. They reflect the inconsistencies between regional carbon emission performances and urbanization, resulting in weak spatial interdependence between them. These results highlight the need to foreground the coordination of environmental protection in the process of advancing urbanization. An examination of the change trend reveals a steady increase in the consistency of carbon emission performances and urbanization in space. Moreover, all of these values exceeded 0, thus reflecting a relationship of positive spatial dependence between these two indicators.

**Fig 8:**
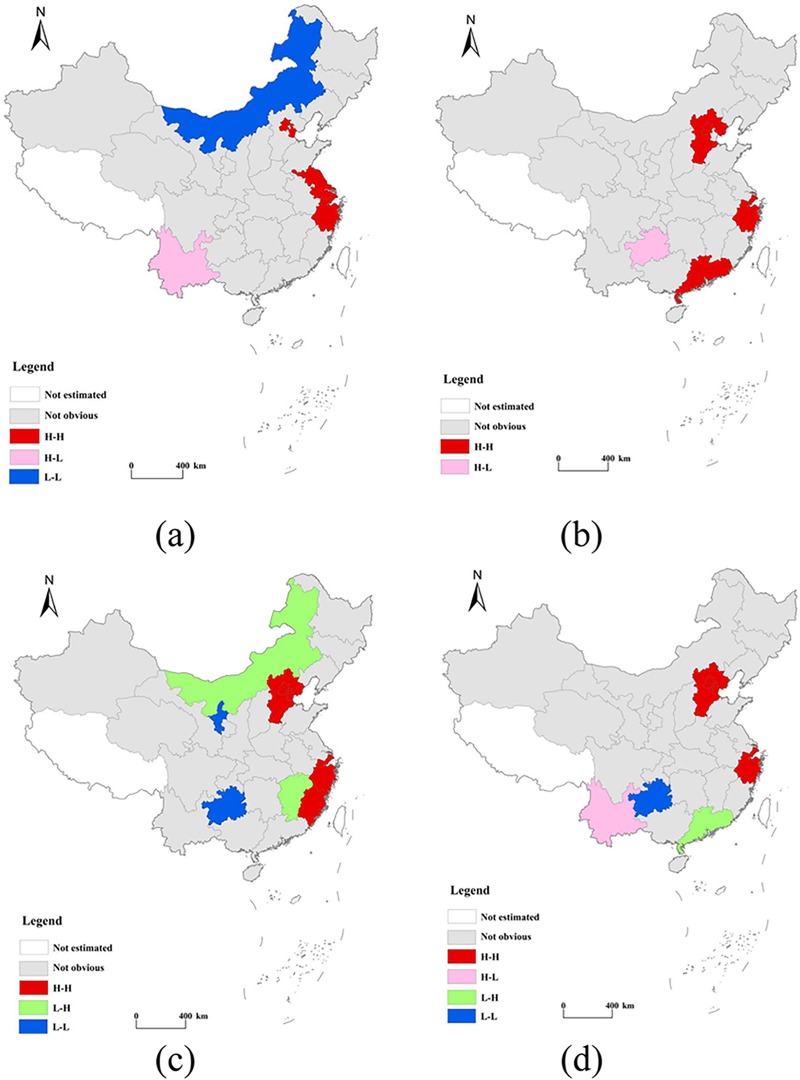
LISA maps depicting carbon emission performances and urbanization of provinces in China in the years 2000 (a), 2005 (b), 2010 (c), and 2016 (d).

We used LISA maps to explore the characteristics of changes in local clustering. Fig 8 shows LISA values at a statistically significant level of 1% in 2000, 2005, 2010, and 2016, respectively. In the LISA maps, a high–high (H–H) region indicates that a province with a high level of urbanization is clustered with adjacent provinces, which also demonstrate high carbon emission performances. Similarly, low–low (L–L) regions denote provinces with low levels of urbanization that are adjacent to those demonstrating low carbon emission performances. High–low (H–L) regions are characterized by provinces with high urbanization levels bordered by those with low carbon emission performances, and low–high (L–H) regions demonstrate the reverse characteristics. Provinces that did not pass the significance test were marked as “not obvious” in the maps.

Fig 8 depicts three hotspot (H–H) regions: the Beijing–Tianjin–Hebei region, the Yangtze River Delta, and Guangdong Province, all of which demonstrate high levels of economic and social development and harmonious environments. Inner Mongolia, Ningxia, and Guizhou, which are limited by natural and historical conditions and a lack of innovative technology were located in an H–L region (“cold” spot). Therefore, the carbon emission performances and levels of urbanization in these provinces were relatively lower. In 2010, Inner Mongolia and Jiangxi were located in a L–H region, as was Guangzhou Province in 2016. Yunnan Province was in a H–L region in 2000 and 2016, and in 2005, Guizhou Province was also part of a H–L region. In light of the positive Global Moran’s I and urbanization values, spatial disparities between carbon emission performance and urbanization were mainly attributed to the agglomeration effect induced by H–H and L–L regions. There was no evident agglomeration effect associated with H–L and L–H regions.

#### Spatial coupling analysis

**Fig 9:**
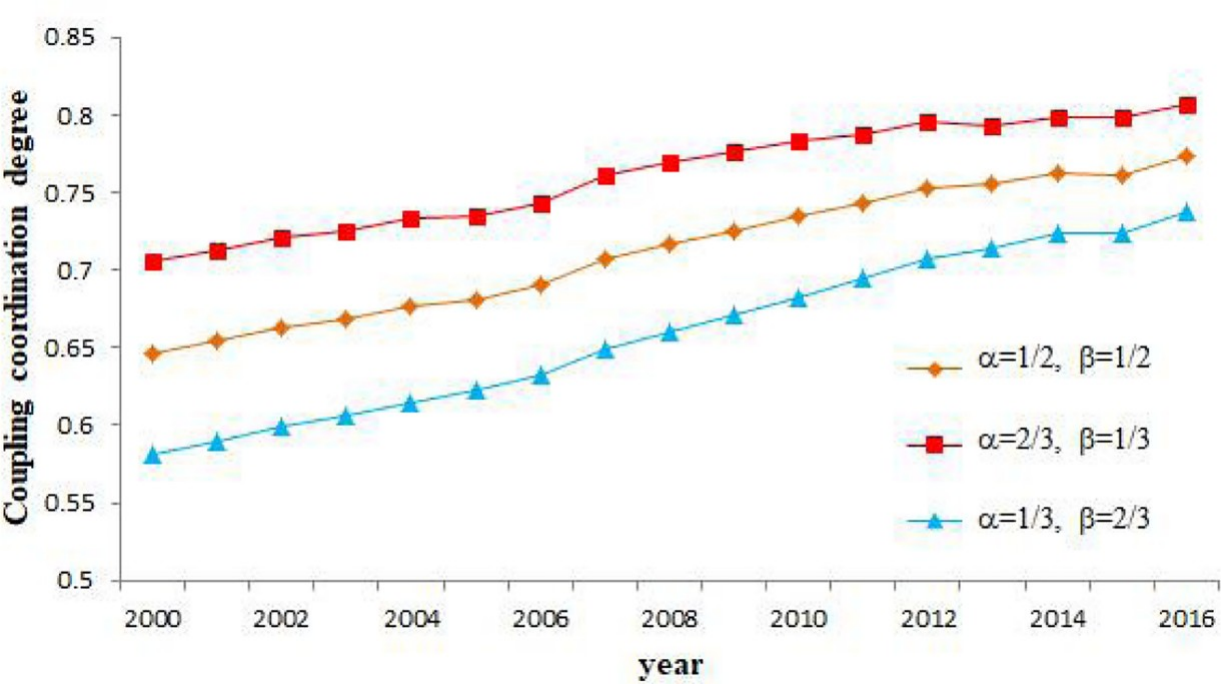
The coordination of coupling between carbon emission performances and urbanization during the period 2000–2016.

To determine the extent to which carbon emission performance and urbanization contributed to the degree of their coordination, we selected three illustrative cases with different carbon emission performance and urbanization values (α = 1/3, β = 2/3; α = 1/2, β = 1/2; and α = 2/3, β = 1/3). As shown in Fig 9, these three cases did not influence the overall change trend. The degree of coordinated coupling between carbon emission performance and urbanization evidenced an overall increasing trend during the study period. The coupling result was better for the values α = 2/3 and β = 1/3 compared with the results for the other two sets of values. This finding implies that the contribution of carbon emission performance exceeded that of urbanization in relation to their coordination.

**Fig 10:**
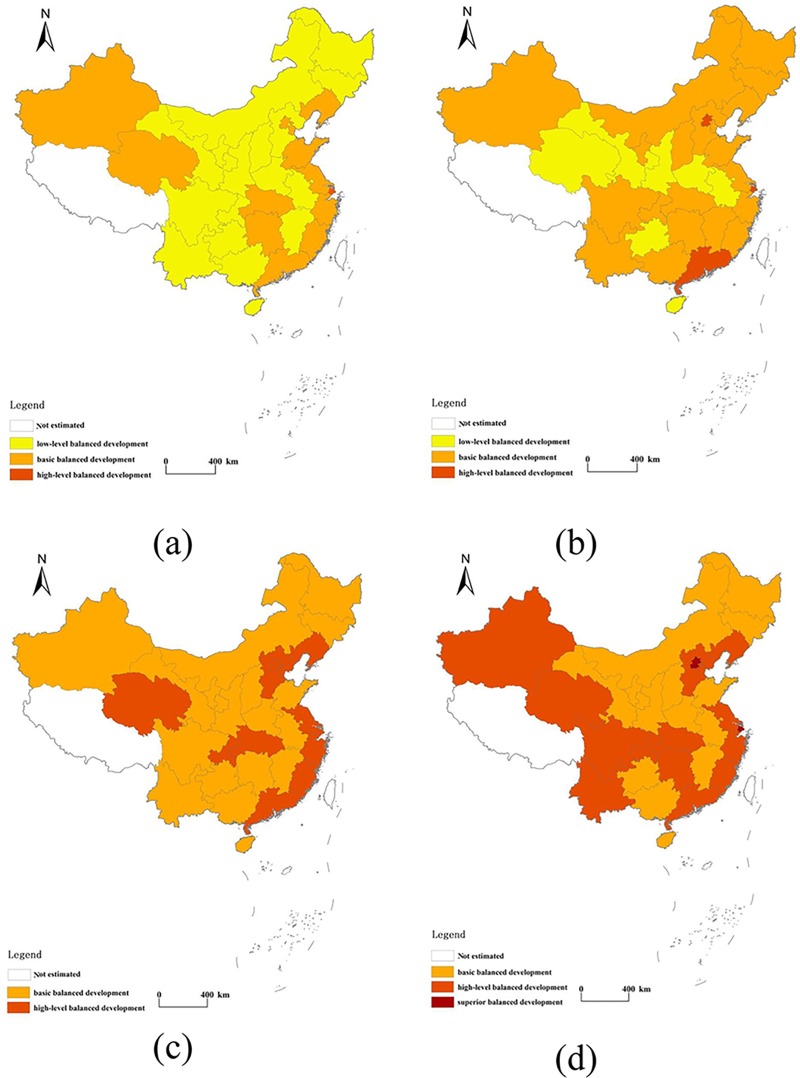
The spatial distribution of coordinated coupling between carbon emission performance and urbanization in 2000 (a), 2005 (b), 2010 (c), and 2016 (d).

The spatial distribution map reveals the trends and spatial distribution characteristics of the coupling relationship between carbon emission performance and urbanization. We plotted the spatial distribution of the coupling coordination for α = 2/3 and β = 1/3. As shown in Fig 10, the coupling of carbon emission performance and urbanization changed between the transition and equilibrium periods from 2000 to 2016. Its average value was 0.807 in 2016, indicating an increase of 14.287% compared with this value in 2000. Of note is the fact that the increase in the degree of coupling gradually spread from the eastern region. Areas with high values of coordinated coupling were strip-shaped, with one cluster located along the eastern coast and the other clusters in the central and southern regions. Beijing (0.898), Shanghai (0.881), Tianjin (0.876), Zhejiang (0.843), and Fujian (0.839) had relatively higher coordination values in 2016, as did Xijiang (0.776), Qinghai (0.798), Sichuang (0.799), and Yunnan (0.753). By comparison, the values of Hainan (0.669), Guangxi (0.683), and Guizhou (0.699) were relatively lower during this period.

## Discussion and conclusions

### Discussion

The aim of urban development is to generate comprehensive benefits for urban residents. To achieve harmonious coexistence requires not only the achievement of optimum urbanization but also improved efficiency of carbon emissions through the application of science and technology during the development process. Our research findings show that while increasing capital and labor inputs has a positive effect on carbon emissions performance, leading to its improvement, excessive energy consumption has a negative effect, which is consistent with the findings of several scholars (e.g., [35, 36]). The findings of our study on urbanization could also provide empirical evidence for a large-scale study. Specifically, our finding of a previously unknown relationship of positive spatial dependence between carbon emission performance and urbanization is a contribution to the literature. However, this relationship varied because of disparities in regional development. Carbon emission performance and urbanization in the eastern region were generally high and evidenced a significant H–H clustering pattern. Given its advantages of relatively advanced socioeconomic development and rapid exchanges of material and energy, the eastern region has the capacity to coordinate the relationship between carbon emission performance and urbanization while continuously improving the former. In addition, the western provinces also demonstrated a relatively strong coupling relationship between carbon emission performance and urbanization. There are two main reasons for this. First, the proportion of carbon emission performance was excessive, and our selected values of α = 2/3 and β = 1/3 indicated that provinces with higher carbon emission performances were more dominant in the western region. In addition, the calculated carbon emission performance reflects the relationship between input and output factors within the production function. In other words, the level of carbon emission performance is more aligned with the relative balance of input and output factors than with the size of the factor values.

In view of our findings, we offer three policy recommendations:

The firstly recommendation entails adaptation to local conditions. We recommend the adoption of reasonable measures to improve carbon emission performance and urbanization that are based on the actual situation within a particular region. There are evident regional differences within China, and the development of cities is not uniform. Thus, although the values of urbanization and carbon emission performance are high in the eastern region, it is still necessary to focus on maintaining their coordination and on curbing the overexpansion of the radiating range. While the carbon emission performances of western provinces were found to exceed their levels of urbanization, maintaining this state in relation to urban development should be prioritized. It is not feasible to pursue GDP blindly while sacrificing the environment. Evidently, such blind pursuit has already happened in, for example, the central region where carbon emission performance and urbanization are not adequately coordinated. Future urban development of this region will require greater investments in the urban environment. At present, many central cities are currently in the process of actively transforming their industrial structure.

A second recommendation concerns popularizing green energy-saving technologies. Our results have shown that energy consumption remains an important constraining factor for the improvement of carbon emission performance. Therefore, policy makers should promote advanced technology and focus on improving the low-carbon economy rather than on merely reducing the total amount of carbon emissions. At the same time, enterprises should be well publicized. The introduction of enterprise production technology is necessary not only for improving economic efficiency but also for improving carbon emission efficiency, representing a win–win situation for the economy and the environment. In addition, policy makers should target high-energy enterprises whose efficiency is low to bring about their transformation. It is necessary for the government to provide increased support, for example, through increased investments, to new tertiary industries.

A third recommendation relates to the provision of guidance for urban residents. The behavior of urban residents has an important impact on carbon emission performance. In cities with advanced urbanization and carbon emission performances, it is necessary to provide residents with guidance to enhance their environmental awareness and inculcate low-carbon living habits among them through their active participation in environmental protection activities and their purchase of green energy-saving products. Moreover, in cities where urbanization and carbon emissions performance are in urgent need of improvement, urban development requires significant investments of labor and efforts to strengthen economic vitality. Therefore, policy makers should attend to the quality of the nonlocal labor force. Free classes and environmental lectures can be organized for this purpose, enabling this group to participate more effectively in urban construction.

In sum, we conducted a spatial analysis of the relationship between carbon emission performance and urbanization in China for the period 2000–2016. However, further analysis is required in the following two areas. First, whereas we highlighted the carbon emission performance of 30 provinces, an analysis entailing a larger number of samples at lower scales, such as cities and counties, would yield further insights. Second, urban development is a long-term process, and the relationship between urbanization and carbon emission performance requires continuous monitoring.

## Conclusions

We estimated carbon emission performance and urbanization values using the directional distance function and separate index systems for the period 2000–2016. The key results of our spatial analysis of the relationship between carbon emission performance and urbanization were as follows.

First, an increase in the capital stock and labor inputs was found to be necessary to improve carbon emission performance. The carbon emission performance of the eastern region was relatively higher, on average, than the performances of other regions. Moreover, the northwestern region evidenced a clear decreasing trend. Over time, the gap in the carbon emission performances among provinces widened. The government should take steps to curb inefficient energy consumption in Hebei, Guangdong, Qinghai, and Xinjiang. The adoption of new technologies and more effective management systems should simultaneously be encouraged and supported in Shanxi, Anhui, Inner Mongolia, Guizhou, Shaanxi, and Ningxia.

Second, the average urbanization value of each province showed an upward trend during the period 2000–2016. The average urbanization value of the eastern region was higher than the values obtained for other regions in China during this period. The urbanization of 30 provinces assumed a “striped” spatial pattern of agglomeration. High urbanization values were concentrated along the eastern coast, with relatively lower values found for the central and western regions.

Third, these two indicators evidenced a relationship of positive spatial dependence, with the strengthening of spatial agglomeration during the period 2000–2016. Global Moran’s I values for carbon emission performance and urbanization in China ranged between 0.146 and 0.255 during the study period. As depicted in the LISA map, H–H and L–L aggregation patterns were predominant. Moreover, carbon emission performance played a greater role than urbanization in their coordination. A further finding was that the average values of the coordination of coupling between carbon emission performance and urbanization showed an upward trend, with an annual increase of 0.85%. Areas with high values of coupling coordination evidenced a strip-shaped pattern of spatial distribution.
